# Even Lower Latency in IIoT: Evaluation of QUIC in Industrial IoT Scenarios †

**DOI:** 10.3390/s21175737

**Published:** 2021-08-26

**Authors:** Fátima Fernández, Mihail Zverev, Pablo Garrido, José R. Juárez, Josu Bilbao, Ramón Agüero

**Affiliations:** 1 Ikerlan Technology Research Centre, Basque Research Technology Alliance (BRTA), 20500 Arrasate/Mondragón, Spain; mzverev@ikerlan.es (M.Z.); pgortiz@ikerlan.es (P.G.); jrjuarez@ikerlan.es (J.R.J.); jbilbao@ikerlan.es (J.B.); 2 Department of Communication Engineering, University of Cantabria, 39005 Santander, Spain; ramon@tlmat.unican.es

**Keywords:** QUIC, Message Queuing Telemetry Transport (MQTT), Industrial Internet of Things (IIoT), Industry 4.0, wireless networks, emulated environment, performance analysis

## Abstract

In this paper we analyze the performance of QUIC as a transport alternative for Internet of Things (IoT) services based on the Message Queuing Telemetry Protocol (MQTT). QUIC is a novel protocol promoted by Google, and was originally conceived to tackle the limitations of the traditional Transmission Control Protocol (TCP), specifically aiming at the reduction of the latency caused by connection establishment. QUIC use in IoT environments is not widespread, and it is therefore interesting to characterize its performance when in over such scenarios. We used an emulation-based platform, where we integrated QUIC and MQTT (using GO-based implementations) and compared their combined performance with the that exhibited by the traditional TCP/TLS approach. We used Linux containers as end devices, and the ns-3 simulator to emulate different network technologies, such as WiFi, cellular, and satellite, and varying conditions. The results evince that QUIC is indeed an appropriate protocol to guarantee robust, secure, and low latency communications over IoT scenarios.

## 1. Introduction

The term Industry 4.0 was originally proposed by the German government in 2011 [1]. It entails a radical transformation, which would greatly impact the way traditional companies and industries operate. In this sense, the underlying “industrial revolution” is based on four main principles: enhanced inter-connectivity, data transparency, decentralized and automated decision processes, and technical assistance.

One cornerstone of this transformation is the so-called Internet of Things (IoT), which enables the connection of industrial elements that are able to collect and process, in real-time, a large amount of data. This would bring, among other functionalities, the capabilities of monitoring systems, exchanging the gathered (and processed) data, and ascertaining the environment [1]. The development of Industry 4.0 services imposes stringent requirements: low latency communications, high reliability and availability, energy efficiency, and security and privacy [2].

Two of the most important requirements for IoTare energy efficiency and low latency. Sensor nodes mostly run on batteries, and in many cases, their replacement might be rather complex or even not possible. Hence, it is of paramount importance to ensure long-life sensor operation, yet their costs should be kept reasonable, since it is expected that massive deployments will be needed to cover large or complex systems. On the other hand, if the information gathered by these devices is used for critical decisions (as was mentioned earlier), latency stands out as one of the key performance indicators. The goal is to enable control procedures in (almost) real-time situations.

The Message Queuing Telemetry Transport (MQTT) protocol [3,4] is one of the most widespread solutions for traditional IoT applications. It has several features—a small code footprint, easy integration, and good performance—that makes it an appropriate solution for these scenarios. MQTT was built on top of the TCP [5] protocol, which offers a reliable end-to-end communication service. On the other hand, it is well known that TCP cannot be adapted at the pace at which technologies are evolving [6], and it indeed has several limitations, including those caused by the ossification of internet protocols. For instance, the performance of TCP over packet erasure networks has been proven to be severely hindered, with a strong increase of the end-to-end delay [7]. Furthermore, TCP does not appropriately cope with the strict delay requirement of some applications [8].

Hence, several solutions have been proposed to tackle the aforementioned limitations. Some of them correspond to TCP modifications and extensions, such as SCTP [9], Real-Time TCP [10], and Network Coded TCP [11]. Other proposals foster a more clean-slate approach, such as QUIC [12], a novel transport protocol running on top of UDP, whose main design principles are: (1) to reduce connection establishment and transport delays; and (2) to improve security standards, with default end-to-end encryption in HTTP-based applications [13].

In this paper, starting from the proposal of a three-tier fog/cloud architecture that leverages better performance for IoT-based services, we study the performance of the integration of Message Queuing Telemetry Transport (MQTT) with QUIC. We carry out an in-depth analysis, based on a novel methodology, which entails real protocol implementations and Linux containers, and emulation techniques within the ns-3 network simulator. We compare the behavior of this combination with that exhibited by the traditional TCP-based solution.

The main contributions of this paper are:

- Integration of a fully operational Message Queuing Telemetry Transport (MQTT) implementation with a QUIC GO implementation.
- All the code that was used to obtain the results presented herewith has been made available in a public git repository.
- Design and implementation of a methodology to assess the performance of the proposed scheme, using Linux containers and llemulation techniques in ns-3.
- Performance analysis of MQTT when used on top of QUIC over various network technologies. using an extensive measurement campaign, and an in-depth comparison with a more traditional approach based on TCP.
- We used peer-to-peer links to emulate different technologies and conditions, and we also assessed the behavior of the proposed schemes considering a scenario where multiple nodes shared the same access.
- We characterized the gain that can be obtained with some of the features included in the QUIC operation, such as 0-RTT.

The rest of the paper is structured as follows: Section 2 discusses related state-of-the-art protocols and points out the main contributions of this work. Section 3 describes the architecture that we are considering, including the things, fog, and cloud, over which we propose using QUIC as a transport protocol. Section 4 sketches the integration of the Message Queuing Telemetry Transport (MQTT) and QUIC protocol in GO language. Section 5 depicts the different configurations that were used to carry out the experiments. Section 6 discusses the results that were obtained after a thorough measurement campaign, comparing the performance of the proposed scheme with a more traditional solution. Finally, Section 7 concludes the paper, highlighting the aspects that we will tackle in our future work.

## 2. Background

QUIC is a transport protocol originally developed by Google Inc. [13], which has been recently standardized by IETF [12]. QUIC RFC documents have been published after the experiments described in this work were concluded. Hereinafter, when we refer to QUIC, we will be specifically talking about the draft version 27, since it corresponds to the particular implementation we used. Besides addressing some of the most relevant TCP limitations for Industrial Internet of Things (IIoT) (for instance, more precise packet acknowledgment and retransmission), it also brings some additional benefits, which might become rather relevant for the targeted scenarios, since it would yield lower latencies, despite offering a reliable and secure service. On the other hand, Message Queuing Telemetry Transport (MQTT) stands out as one of the most popular application protocols to tackle the requirements of Industrial Internet of Things (IIoT) networks and services. This section introduces both MQTT and QUIC protocols, to better understand the motivation of their combination and its impact in the IIoT realm.

### 2.1. MQTT

Message Queuing Telemetry Transport (MQTT) [3] is a lightweight application protocol which follows the traditional publish-subscribe model. It has become a very popular solution within a rather broad range of industries to connect low-power devices, since it benefits from a small code footprint and does not require large bandwidths. IBM launched version 3.1.1 in 2014, which was afterwards standardized by International Organization for Standardization (ISO) and Organization for the Advancement of Structured Information Standards (OASIS). Although more recent versions have been also standardized (for instance version 5.0 [4]), the one fostered by IBM is still the most widely used.

MQTT defines three different roles: subscriber, publisher, and broker. Publishers are small sensors, which gather information and publish it into a common register (broker), which keeps information belonging to specific topics. The subscribers access the data provided by the publishers. To do so, they subscribe to a number of topics, and they then receive all messages sent by any publisher on those particular topics.

Both publishers and subscribers take the client role in the underlying network topology. On the other hand, the key entity of MQTT is the broker server, which manages the subscriptions and the consequent information delivery to the interested nodes. Hence, all messages published in the network are first sent to the broker, which takes care of forwarding the information to the corresponding subscribers (according to their preliminary registrations). Although clients only interact with a single broker in the current Message Queuing Telemetry Transport (MQTT) specification, the network may contain several brokers, which would actually exchange data among themselves, based on their subscribers’ topics.

One of the main advantages of MQTT is that it isolates the operation of data producers (publishers) and consumers (subscribers). This strongly facilitates the implementation of such functionalities in low computational devices, which can interact by exploiting the publish–subscribe scheme. In this sense, since publishers send new content whenever it becomes available, there exists a temporal decoupling between a node’s interest and the publication of such information.

### 2.2. QUIC

**Figure 1:**
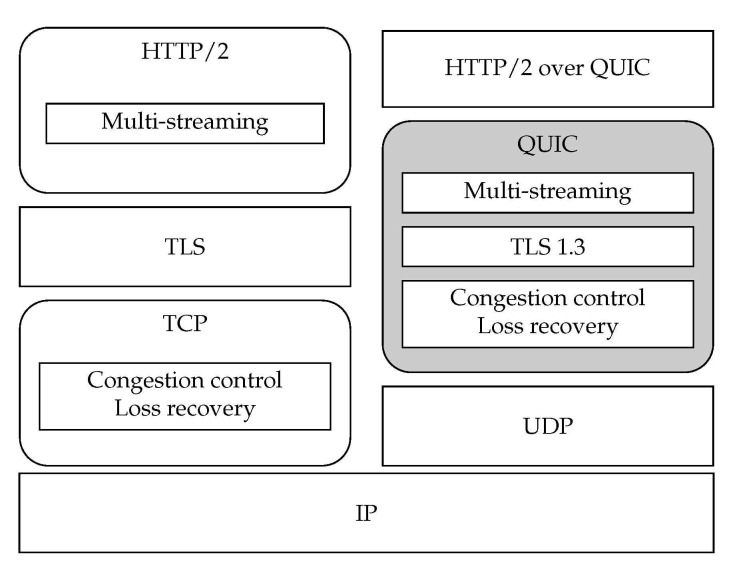
Traditional architecture of the transport layer TCP/TLS vs. the QUIC protocol stack.

QUIC addresses two of the main challenges of today’s web traffic: (1) latency reduction, for a better user experience; and (2) securing the communications (end-to-end payload and header encryption [14,15]), as the Internet is clearly shifting towards more secure web traffic [13]. Figure 1 depicts the QUIC protocol stack, comparing it with the traditional TCP/TLS approach, when used to transport HTTP traffic.

**Figure 2:**
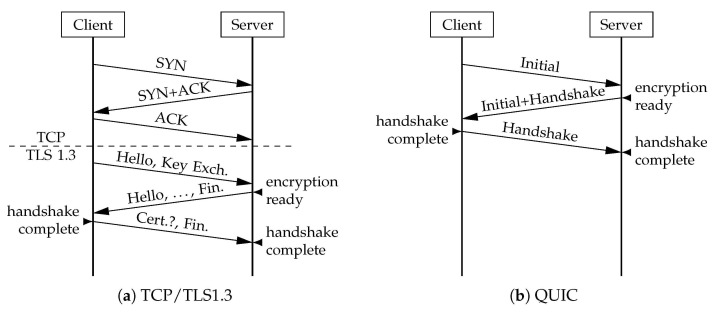
Establishment of TCP (**a**) and QUIC (**b**) with version 1.3 TLS.

QUIC established a secure connection in 1 Round Trip Time (RTT) with Transport Layer Security (TLS) protocol version 1.3. This was achieved by combining QUIC and TLS handshakes. TLS 1.3 connection establishment could be further reduced to 0 RTT if the endpoints previously established a communication [16], a functionality that QUIC also integrates [15]. Hence, data can be sent before a new handshake is repeated in 0-RTT packets. On the other hand, legacy TCP 3-way handshake allows an endpoint to send data only after 1 RTT [5]. Furthermore, TCP alone does not encrypt its payload, and additional protocols, such as TLS, are required to establish a secure connection. Although TLS 1.3 adoption is continuously increasing, TLS version 1.2 is still widely used [17], and its connection establishment takes no less than 2 RTT [18]. Thus, the resulting secured TCP connection establishment takes either 3 RTT with TLS 1.2, or between 2 and 1 RTT with TLS 1.3. Figure 2 compares TCP + TLS 1.3 with QUIC connection establishment.

The information within a QUIC connection is organized in streams, enabling QUIC to prevent delays caused by head-of-line blocking. When a packet is lost, only streams having data in such packets are blocked while waiting for a retransmission, but other streams can continue [14]. QUIC is also able to reduce the overall latency thanks to its loss detection algorithms, including “Early Retransmits” and tail loss probes [19].

The main difference among TCP loss detection mechanisms is that QUIC does not retransmit lost packets with the same packet numbers. QUIC uses acknowledgment-based detection with a probe timeout to ensure acknowledgments are received. QUIC loss recovery mechanisms are applied in the following scenarios [19]:

- If the packet is not acknowledged and it was sent before an acknowledged packet and one of the following conditions is met:
  - Either it was sent with an expiring packet time of 9/8⋅RTT;
  - Or its packet number is three times smaller than the last acknowledged packet.
- If the probe timeout is reached, from the moment the packet was sent, and a subsequent packet is not acknowledged.

QUIC encrypts its payload and protects its headers not only to ensure communication security [14], but also to prevent protocol ossification [13]. At the time of writing, TCP is not straightforward to update, due to the vast deployment of middleboxes, which have been implemented to optimize TCP traffic [6]. Until these middleboxes are updated, they discard all new TCP segments. On the other hand, QUIC packets are seen as UDP payloads, and so their formats, regardless of the version, are invisible to such intermediate entities, ensuring safe interactions with them. QUIC is intended to be deployed in user-space, meaning different applications (e.g., various browsers) at the same node could actually benefit from the QUIC features they need—for instance, managing computational resources or setting logging levels as required. Despite this initial user-space implementation objective, QUIC could be embedded into the kernel too, with the goal of boosting its performance [20].

Although the updating process would be easier with QUIC, not all devices could simultaneously change to a newer version, leading to the coexistence of multiple QUIC versions. Correct operation is ensured with a version negotiation mechanism, which allows the endpoints to agree on the version they will use, before the QUIC connection is established. This feature not only simplifies protocol updates, but also allows customizing QUIC with extra features. The endpoints might even share these features as plugins [21].

### 2.3. QUIC and the IoT Paradigm

Even when QUIC was still under development, some researchers had already integrated it in Internet of Things (IoT) protocol stacks [22,23]. However, we have identified that there do not exist many works evaluating QUIC in IoT scenarios.

Liri et al. considered QUIC as an IoT protocol, comparing it with IoT specific alternative solutions such as CoAP and MQTT [24]. They proved that QUIC, before it was completely specified, was outperformed by CoAP [25]. However, its behavior is comparable to MQTT in lossy and disruptive environments. The authors suggested that a more streamlined version of QUIC could be a potential request–response IoT protocol alternative to CoAP.

Kumar and Dezfouli evaluated Google QUIC’s performance in IoT scenarios [26]. They defined a testbed with Raspberry Pi 3B devices where MQTT over QUIC and TCP performance was assessed. Their evaluation focused on connection establishment packet overheads, latency over lossy links, usage of computational resources in the presence of connection impairments, and performance drops due to connectivity breakdowns. They concluded that QUIC outperforms TCP in multiple aspects. They also identified possible improvements that should be included for its use in IoT scenarios.

Lars Eggert studied the feasibility of implementing QUIC in constrained IoT devices [27]. He used more limited devices than those used by Kumar and Dezfouli [26]: Particle Argon and ESP32-DevKitC V4. Since the main objective was to enable the operation of QUIC in low-capable devices, some of the original QUIC features, likely to be impractical for IoT, were changed, or even removed, to reduce the required memory usage. Eggert concluded that QUIC is indeed a practical alternative to be considered for IoT edge devices.

As the QUIC standardization process has recently concluded with the publication of the corresponding RFC [12], we foresee a renewed interest from various fields. As a follow up in our initial evaluation, which is reported in [28], Saif and Matrawy further assessed QUIC feasibility for IoT scenarios, combining it with HTTP/3 [29]. They also understand that delay should be considered as the key performance indicator. In opposition to what we consider herewith, they did not include in their evaluation different technologies, nor packet erasures over the wireless accesses.

Finally, it is worth highlighting that this paper amply broadens the analysis discussed in [28], where we preliminary assessed QUIC performance over IoT scenarios. We consider more complex setups, based on the fog/cloud architecture that we sketch in Section 3, where devices use Message Queuing Telemetry Transport (MQTT) on top of both QUIC and TCP to establish the corresponding communications. We study the overall delay, comparing the performance of both transport protocols over error-free and packet-erasure channels with various Frame Error Rate (FER) values, considering WiFi, cellular, and satellite technologies. We also evaluate the performance exhibited by QUIC and TCP over shared channels. We have also included an additional QUIC feature (0RTT) and studied its benefits, by using an appropriate traffic pattern.

## 3. QUIC-Based IIoT Architecture

In our evaluation we consider a three-tier architecture to deploy IIoT services, which includes the following levels [30]:

- Things. These are the entities that perform measurements to monitor the environment (sensors) or take actions (actuators) to adapt the system’s behavior and its operation. They normally use wireless links to connect with the rest of the architecture.
- Fog. The traditional cloud approach (where the information gathered by the things is sent to a node that is deployed in centralized servers) has two major limitations. First, it could incur long delays when reaching nodes from the things layer. Secondly, if a fully centralized approach is used, the system might suffer from scalability issues, which might hinder its usefulness. A potential solution would be the distribution of the computational burden among various nodes, bringing them closer to the things. Hence, the use of edge computing, where the servers are next to access elements (base stations, access routers, etc.), is currently receiving interest from the scientific community.
- Cloud. In IIoT, it might be of great relevance to react as quickly as possible to changes or events. The use of artificial intelligence and machine learning enables the system to make automated decisions, reducing the response time that would be required by traditional human operators. The virtualization and software-defined networks (SDN) paradigms allow the deployment of intelligent agents that leverage this approach in cloud nodes.

The rapid proliferation of IoT in different areas, particularly in so-called Industry 4.0, is not matched by the pace at which the actual solutions evolve. Message-based protocols, such as MQTT and COAP, which usually rely on traditional client/server architectures, are probably not the most appropriate choice for systems with multiple fog and cloud servers. In particular, some previous papers have analyzed how the three-tier architecture might bring important benefits in terms of operation cost (for distributed storage services), and/or performance, by considering the time required to promote certain decisions [31,32].

However, the applicability of this architecture might be hindered by the already strong adoption of message-based protocols (MQTT and COAP). In this sense, many of the potential benefits of the proposed three-tier architecture imply the use of rather novel techniques: multi-path communications, coding techniques, and other features. Traditional communications, usually TCP, do not support such functionalities.

For instance, multi-path communications can provide interesting benefits to the IIoT realm, since they allow the establishment of different paths to exchange information between the two communication edges, or to naturally send the information to a set of nodes (fog/cloud) that would afterwards cooperate.

In IIoT, when things are connected to more than one access element, the use of multi-path could yield better performance and/or reliability, by simultaneously exploiting the various available paths [33]. Furthermore, in fog/cloud scenarios, it would be interesting to promote distributed storage or decision systems, and communications might have multiple sources (downlink) or destinations (uplink). Another potential benefit of multi-path communications in IIoT scenarios would be a more appropriate means of congestion management (both in terms of computational resources at edge/cloud nodes or communication resources at various links).

We argue that, in order to promote the aforementioned architecture, the use of TCP might not be an appropriate choice, and we thus propose to use QUIC since it does not only bring latency improvements, as we evaluate herewith, but it also allows easier deployment of its customized versions, thanks to its middlebox independence. One of the most comprehensive studies in extending QUIC with extra features was [21]. In spite of these clear benefits, there are not many works that have actually analyzed the feasibility of using traditional message-based protocols (such as MQTT) over QUIC, which shall be a first step to enable the use of this transport protocol in IIoT scenarios.

## 4. Implementation

In order to carry out the analysis of QUIC as a transport protocol for IoT, we used a QUIC implementation in GO language, quic-go (https://github.com/lucas-clemente/quic-go (accessed on 10 April 2020), version 0.15.1.), which follows the IETF QUIC draft version 27. At the time of writing, quic-go implements and facilitates the use of the newly released RFC 9000 [12].

**Figure 3:**
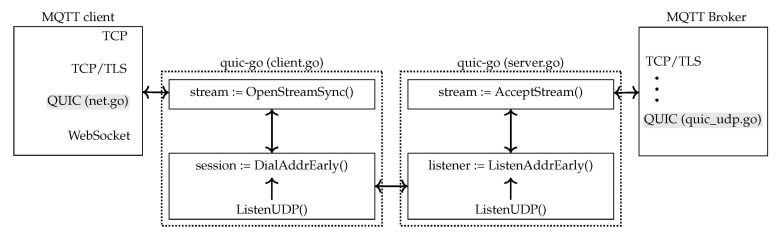
Implementation scheme of QUIC socket in MQTT.

The MQTT client and the server broker are based on open-source Eclipse Paho (https://github.com/eclipse/paho.mqtt.golang (accessed on 10 April 2020), version v1.2.0.) and VolantMQ (https://github.com/VolantMQ/volantmq (accessed on 10 April 2020), version v0.4.0-rc6.), respectively. Both of them are implemented in GO and support MQTT 3.1 and 3.1.1. Eclipse Paho builds a library that enables applications to connect to VolantMQ broker using TCP, TLS or WebSocket. Both projects are based on GO 14.0 version, and they are open-source, which allowed us to tweak them to use QUIC, which was also developed with the same GO version. Figure 3 illustrates the changes that were made over the legacy implementations of both *net.go* and *quic_udp.go*.

In the client case, all the functionalities at the transport layer, i.e., opening and closing connections, or sending and receiving packets, are managed from the *net.go* interface. Based on this interface, the integration of the QUIC socket involves minor changes in the original code supporting TCP, TCP+TLS, QUIC, and WebSocket connections. In order to enable the use of QUIC connections, the *net.go* interface calls, from *quic-go*, the *client.go* interface. Due to the 0-RTT operation, we use the *DialAddrEarly* function to keep “early” data before the handshake completes. This MQTT client, which can be used over the QUIC open-source implementation, has been made available, and can be accessed at a public git repository (https://github.com/pgOrtiz90/paho.mqtt.golang (accessed on 10 April 2020)).

The broker implementation is also open source, and publicly available in a git repository (https://github.com/fatimafp95/volantmq_2 (accessed on 10 April 2020)). The transport level functionalities are implemented using *transport/conn.go* interface. The incompatibilities and restrictions between the connection interfaces offered by TCP and QUIC, this MQTT server only supports QUIC connections. We integrated the *quic_udp.go* interface, which calls the listener from the *server.go* interface of *quic-go*. The listener function is *ListenAddrEarly*, to enable 0-RTT on the server broker side. This function enables a client that has previously connected to the broker to use the information stored in the cache of that session. Therefore, re-establishing the connection does not require re-negotiating all parameters, so the data exchange happens before the handshakes actually completes.

Figure 3 depicts how the complete communication is created. The broker is listening on the QUIC socket through the *ListenAddrEarly()* function. The client establishes a new QUIC connection to the server, with the 0-RTT operation, through *DialAddrEarly()*. After the session is created, the client opens a stream by means of *OpenStreamSync()*, and the broker accepts it with the *AcceptStream()* function.

## 5. Environment Setup

**Figure 4:**
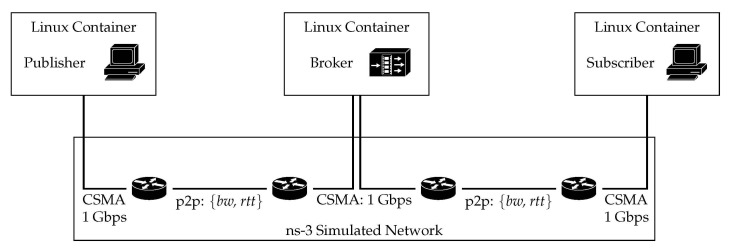
Scenario A. Three Linux containers running different MQTT roles. From left to right: publisher client, broker server, and subscriber client. The broker is connected to each client over a P2P link, which emulates different wireless access technologies.

Experiments were performed using the discrete-event network simulator for internet systems ns-3 (https://www.nsnam.org/ (accessed on 10 April 2020), version 3.24). It provides the possibility to use virtualization techniques to connect traffic from real applications by running LXC Linux containers (LXC version 3.0.3, Ubuntu 14.04 images) over a simulated network. We have deployed an emulated platform, based on the scenario depicted in Figure 4, which hereinafter will be referred to as Scenario A. It entails three Linux containers, which are connected through ns-3. Each network emulates various wireless technologies, considering two parameters: bandwidth and delay. End containers run the client applications, publisher and subscriber, while the middle container runs the broker server.

Linux containers are considered ghost nodes by the ns-3 instance. A CSMA network connects the three of them to simulated routers, with rather high capacity, to enforce the bottleneck to be at the point-to-point link (P2P). These routers are connected over two links, which emulate different technologies. These are configured using the parameters depicted on Table 1. Bandwidth, delay, and loss rate are modified according to various configurations, mimicking different networks and conditions. Even though there exists evidence that cellular networks keep buffer sizes larger than the bandwidth-delay product (BDP) of the path, which might lead to bufferbloat effects [34], we set all buffers to be one BDP.

In a second scenario, we changed the connection between the publisher and the broker, and we used WiFi to connect Linux containers. This feature allowed us to analyze the performance of the combination of MQTT and QUIC over a shared channel. In this case, the connection between broker and subscriber emulates an end-to-end connection over a high bandwidth network. In order to facilitate the execution of systematic and repetitive experiments, mimicking the conditions that were used for the previous scenario, we have modified the error model originally implemented in ns-3, allowing us to precisely control the frame error rate, regardless of the interference model. Furthermore, we have run experiments modifying the maximum number of retransmissions, which are defined in IEEE 802.11 MAC protocol [35] to assess the potential impact of this parameter on the application layer. By disabling this functionality, we can, for instance, ensure that network reliability mostly depends on the transport protocol.

**Figure 5:**
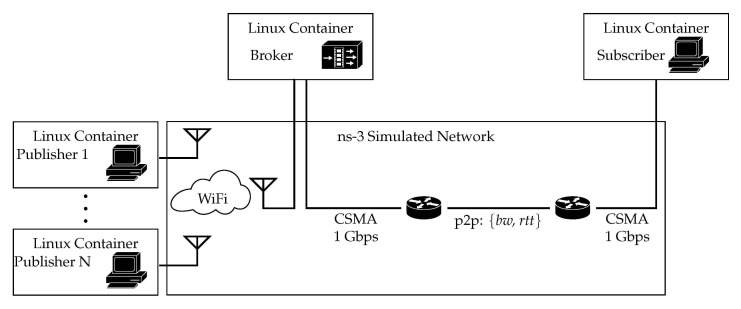
Scenario B. Various publishers connect to the broker over a shared WiFi network.

We define Scenario B based on an ad-hoc wireless network that connects various Linux containers, acting as MQTT publishers, to the broker server over a shared WiFi network. As mentioned earlier, the broker and the subscriber are connected over a P2P link, whose characteristics are fixed to emulate a high bandwidth end-to-end network. Figure 5 illustrates this setup.

## 6. Results

**Figure 6:**
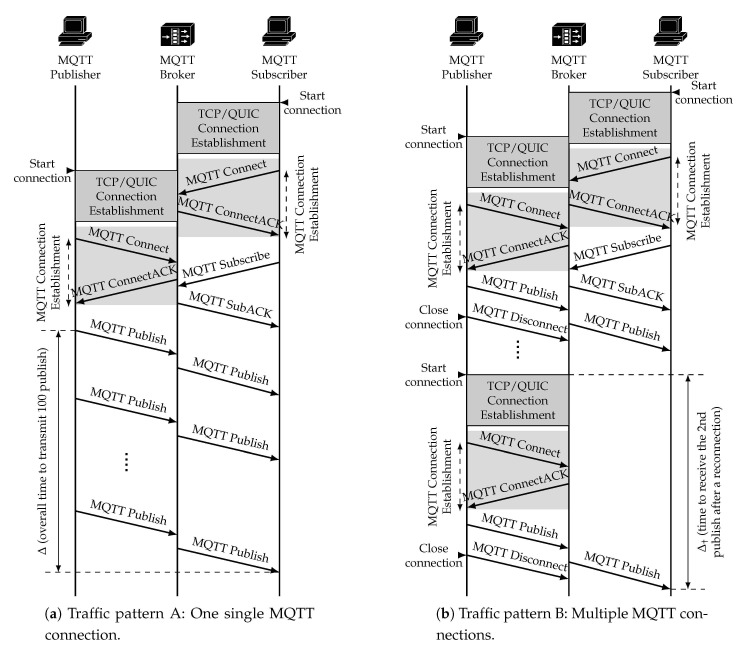
Traffic patterns used during the experiments.

In this section, we discuss the performance of MQTT over QUIC, comparing it with the performance exhibited by a more traditional approach which uses TCP and TLS. We discuss results obtained during an intensive experiment campaign carried out over the scenarios that were described in Section 5. We go beyond the analysis that we carried out in [28]: (i) we consider a shared channel and multiple publishers, (ii) we assess the benefits of additional QUIC features, such as the 0-RTT option, and (iii) we also evaluate the impact of modifying the characteristics of the connection between the broker and the subscriber. Section 6.1 focuses on the results obtained in Scenario A (Figure 4), whereas Section 6.2 discusses the ones obtained in Scenario B (Figure 5). In addition, we used two different traffic patterns, which are depicted in Figure 6. In the first one we measured the overall time required to send a number of publish messages between publisher and subscriber, whereas the second pattern allowed us to assess the benefits of the 0-RTT feature of QUIC, by measuring the time elapsed between the publisher opening a connection to sending a publish message, and the subscriber getting it.

In order to fairly compare the performance of QUIC and TCP, we use their absolute completion times, according to the two parameters defined in Figure 6. We also use the completion ratio ξ metric, which is defined as follows:

(1) $$\xi =\frac{T_{\mathrm{QUIC}}}{\bar{T_{\mathrm{TCP}}}}$$

where $T_{\mathrm{TCP}}$ and $T_{\mathrm{QUIC}}$ are the TCP and QUIC completion times, respectively. These refer to ∆ (Figure 6a). This ratio directly compares QUIC and TCP completion times. Values of ξ lower than 1 yield that QUIC outperforms TCP, since the required time to transmit all packets would be shorter. Furthermore, as we divide by the average completion time of TCP, the variability of the corresponding results reflects that seen with the QUIC protocol.

### 6.1. Scenario A: Single Publisher

We first show the scenario depicted in Figure 4, wherein we used traffic pattern A (cf Figure 6a) to asses the performance of QUIC and TCP. Both MQTT clients used one single MQTT connection, which was not closed throughout the experiment, to send 100 short messages from publisher to the broker, which forwarded them to the subscriber. We forced the MQTT service to follow stop&wait behavior, so the *i*th packet was only sent after ensuring the subscriber got the previous one. We measured the time required to correctly receive the 100 messages (∆ in Figure 6a). Furthermore, to ensure statistically tight results, we repeated this experiment, for each network configuration, 50 times.

**Figure 7:**
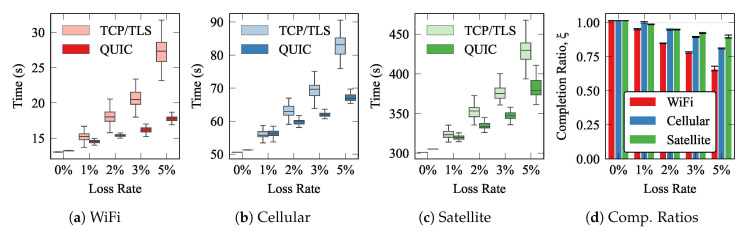
MQTT over QUIC and TCP completion times for traffic pattern A.

Figure 7 depicts the whisker plots of the completion times, and the completion ratios for each technology. Each whisker shows the median (0.5-percentile), as a horizontal line within the boxes, and the 0.25 and 0.75 percentiles (lower and upper box limits, respectively). Last, the upper and lower whiskers correspond to the 1.5 IQR. The results yield that QUIC had far better performance than TCP, especially over networks with low RTTs (WiFi) and with high loss rates. When there was not any loss in the underlying networks (loss rate 0%), we can see that the performance of QUIC was slightly worse than that exhibited by TCP. This is because QUIC was designed to outperform TCP when the conditions of the network are not ideal. For that purpose, QUIC promotes a more complex and advanced flow management scheme, which requires adding some extra overhead. Hence, when the conditions are ideal, the overall delay is higher. Additionally, we can see that when the RTT was higher, the gain that QUIC created was less noticeable, especially for satellite links. In any case, the results evince that QUIC clearly outperforms the traditional approach, not only in terms of the average delay, but also by exhibiting far less variability, which is also a relevant advantage, since large jitters could hinder the behavior of real-time services.

One of the main characteristics promoted by QUIC is the lightweight connection establishment. In order to analyze this improvement compared with the traditional TCP/TLS scheme, we used traffic pattern B, as depicted in Figure 6b.

**Figure 8:**
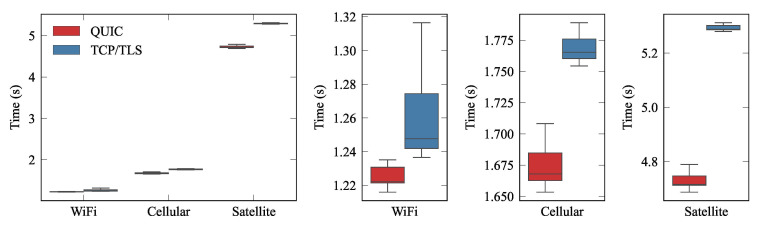
0-RTT approach with three nodes: publisher; broker; subscriber. The left box plot depicts completion times for the emulated technologies. The other box plots provide enlarged views of the first one.

In each experiment, the publisher established an initial connection and then the connection was restablished whenever a message was published. Each experiment was repeated 50 times. We measured the time since the second connection was initially established on the publisher side until the corresponding message was received by the subscriber (${∆}_{†}$; cf Figure 6b). This configuration was used to assess how QUIC is able to strongly reduce latency when resuming a connection. Figure 8 shows the results for 0-RTT tests using the setup depicted in Figure 4. The networks were configured with the parameters shown on Table 1. We can see how the QUIC 0-RTT mechanism strongly reduced connection latency compared to TCP/TLS, in all configurations. Furthermore, QUIC exhibited more stable performance (with much less variability) than the traditional scheme.

**Figure 9:**
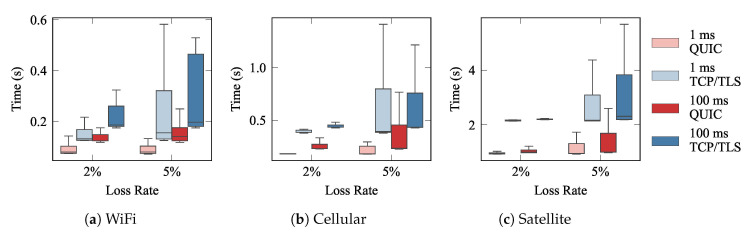
The publisher–broker network was fixed with the parameters of Table 1 and the broker–subscriber link with BW = 10 Gbps and RTT = 100 ms.

One of the reasons to introduce a fog layer between the end-devices (things in our case) and the cloud is the large distance between the latter two, which directly hinders the latency. The performance improvement achieved with QUIC is not expected to change significantly if the conditions of the fog/edge to cloud connection change. We verified this by repeating the completion time evaluation but changing the characteristics of the link connecting the broker, which is representing fog layer, and the subscriber, the cloud. In this experiment, the p2p link emulated a 10 Gbps connection with RTT values of 1, and 100 milliseconds (ms), to reflect a broad range of conditions. The results are depicted in Figure 9. QUIC still offered a noticeable reduction of latency, and led to a rather more stable performance, which was observed in lower result variability.

### 6.2. Scenario B: Multiple Publishers on Shared Access Link

The following results are based on Scenario B, in which we used both traffic patterns. We considered several MQTT clients connected to the broker over a shared WiFi network. This setup mimicked a wireless sensor network in a three-tier (fog/cloud) architecture (as the one sketched in Section 3), where many edge-devices generated data to be processed at the fog layer, and if necessary, to be sent to the cloud (subscriber role) through a wired link with low RTT (25 ms) and high bandwidth.

**Figure 10:**
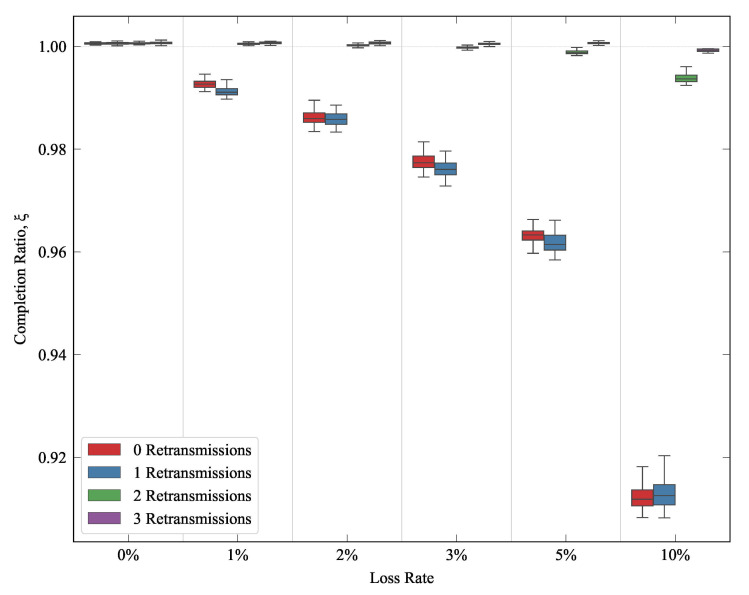
Completion ratios with the numbers of WiFi retransmissions.

As was previously introduced in Section 5, IEEE 802.11 standard (WiFi) specifies a maximum number of retransmission attempts for any frame that is lost [35]. On the other hand, there could be IoT use cases where publishers would be connected through non WiFi wireless shared access medium, without any retransmission at all. In order to asses QUIC and TCP performance in such scenarios, and to decouple the results from the MAC layer operation, we used different maximum retry values (0, 1, 2 and 3) in the WiFi network, when studying the delay for traffic pattern A. The experiment was executed 50 times for each configuration (error rate and retransmission value). Each run entailed the transmission of 100 MQTT packets from the publisher to the subscriber. Figure 10 illustrates the resulting download completion ratio, showing how QUIC could reduce the latency over wireless shared access media. We can also see that this gain is less relevant when the number of retransmissions is larger. We can thus infer that, when this feature is enabled, it would be enough to cope with such eventual losses (especially if the loss rate is low), and the gain of QUIC is thus less relevant. In this sense, we can see the same behavior that was discussed earlier for Figure 7. When the loss perceived by the transport layer was 0%, the performance of TCP was slightly better than that shown for QUIC (ξ>1). When the link loss rate increased, and/or fewer MAC retries were used, the transport layer suffered losses, and QUIC thus yielded a lower delay than TCP.

**Figure 11:**
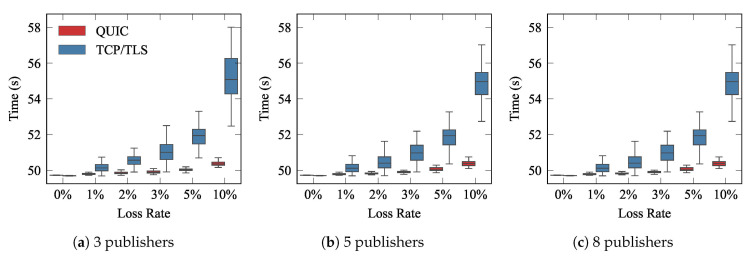
The performance of MQTT over QUIC and TCP/TLS in a shared channel with traffic pattern A.

As mentioned earlier, Scenario B was proposed to emulate the case in which various edge-devices generate data, which is sent to the broker over a shared WiFi network. It is foreseen that the more publishers compete for a medium, the longer the overall communication delay will be. We evaluated QUIC and TCP performance with 3, 5, and 8 publishers, transmitting traffic pattern A, and we further assumed the subscriber was subscribed to the information provided by just one publisher. Figure 11 illustrates the completion times corresponding to the whole transmission of the information that the subscriber was expecting. We measured the ∆ parameter that was introduced in Figure 6a. As can be seen, QUIC clearly outperformed TCP/TLS in all cases, showing, in addition, more stable behavior, exhibiting less variability.

It can also be seen that there was no clear among between the results obtained with different numbers of publishers. We argue that a greater difference would be observed with a larger number of things competing for the shared link. A stronger impact would actually be seen with higher traffic load, i.e., longer and/or more frequent messages, since they would induce more competition on the WiFi link and likely lead to a bigger difference in the results depicted in Figure 11.

**Figure 12:**
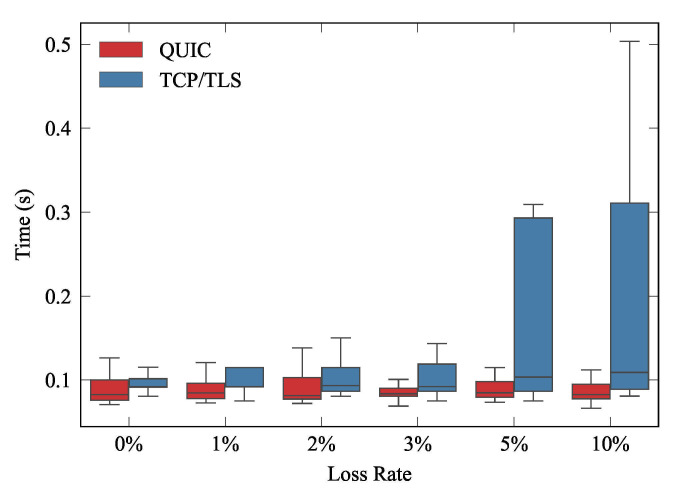
0-RTT approach on a WiFi network with 8 publishers.

Finally, to validate the 0-RTT approach of QUIC, we ran the experiment for traffic pattern B, with eight publishers. This experiment was executed 50 times for each loss rate specified in Table 1, thereby ensuring statistically tight results. The results shown in Figure 12 evince that QUIC is able to reduce latency when a client resumes its connection with the server. It addition, when the channel conditions worsened, we can see that QUIC was able to yield much more predictable behaviors.

## 7. Conclusions

In this paper we have proposed QUIC as an alternative to the traditional TCP/TLS transport means to support latency-critical industrial applications over MQTT.

We have developed a GO-based implementation of MQTT over QUIC, and we have carried out an extensive analysis of its performance in emulated IIoT environments. By exploiting the ns-3 network simulator, we configured different network technologies, characterized by different bandwidth and delay tuples. In addition, ns-3 allowed us to emulate a more realistic environment, where various MQTT clients published messages (i.e., things) over a shared WiFi network to a broker (fog), which consequently relayed the information to a subscriber (cloud), over a P2P network that mimicked the connectivity between fog and the cloud. Thanks to the simulator and its ability to connect Linux containers, we thoroughly assessed the performance of using QUIC as a transport protocol for MQTT traffic, and we compared it with the traditional MQTT/TLS/TCP stack.

Despite the implementations’ restrictions, we developed two complementary scenarios to analyze the behavior of MQTT with both QUIC and TCP. Since the envisaged services have stringent requirements in terms of latency, our evaluation focused on the delay. In this sense, we saw that QUIC clearly outperforms TCP, especially for connections with low RTT and high packet erasure rates. On the other hand, we have also ascertained the benefits of the 0-RTT scheme that QUIC promotes. The results show a clear reduction of latency upon connection establishment. QUIC also yields a more predictable behavior, with much less variability in the results. Finally, we have evaluated QUIC on shared channels, by having multiple things (Linux containers) sending publish messages to the broker over a shared WiFi network. The results evince that QUIC yields a good behavior over shared environments.

All the code that was used to carry out the experiments described in the paper has been made available as public git repositories.

In our future work, we will exploit the methodology we have proposed in this paper to further broaden the evaluation of QUIC as a transport protocol for IIoT environments. We will integrate some of the additional features that are, at the time of writing, under development, such as multi-path. Another QUIC characteristic that was not integrated in the implementation we used is piggybacking, and this might yield some performance improvements, so it might be of interest to assess its behavior. We will also study the impacts of having different traffic patterns and various congestion control algorithms. The platform that was developed in this work would facilitate the evaluation of all these features.

## Figures and Tables

**Table 1:** Network parameter ranges for different wireless technologies.

	NetType1	NetType2	NetType3
	**WiFi**	**Cellular**	**Satellite**
Capacity [Mbps]	20	10	1.5
RTT [ms]	25	100	600
Loss Rate [%]	[0, 1, 2, 3, 5, 10]		

## Data Availability

The data reported in this study are available on request from the corresponding author.

## Abbreviations

The following abbreviations are used in this manuscript:

UDP	User Datagram Protocol
TCP	Transmission Control Protocol
TLS	Transport Layer Security
MQTT	Message Queuing Telemetry Transport
IoT	Internet of Things
IIoT	Industrial Internet of Things
CoAP	Constrained Application Protocol
RTT	Round Trip Time
BW	Bandwidth
FER	Frame Error Rate
P2P	Point-to-point
CSMA	Carrier Sense Multiple Access
QoS	Quality of Service
OASIS	Organization for the Advancement of Structured Information Standards
ISO	International Organization for Standardization
